# Effects of lingual frenotomy on breastfeeding and electrical activity of the masseter and suprahyoid muscles

**DOI:** 10.1590/2317-1782/20232021262

**Published:** 2023-04-21

**Authors:** Hellen Kalina Medeiros Porto de Souza Santos, Daniele Andrade da Cunha, Rodrigo Alves de Andrade, Midiane Gomes da Silva, Ana Cláudia da Silva Araújo, Roberta Lopes de Castro Martinelli, Hilton Justino da Silva

**Affiliations:** 1 Programa de Pós-graduação em Saúde da Comunicação Humana, Universidade Federal de Pernambuco - UFPE - Recife (PE), Brasil.; 2 Universidade Federal de Pernambuco - UFPE - Recife (PE), Brasil.

**Keywords:** Infant, Newborn, Lingual Frenum, Ankyloglossia, Electromyography, Breast Feeding, Speech, Language and Hearing Sciences, Lactente, Recém-Nascido, Frênulo Lingual, Anquiloglossia, Eletromiografia, Aleitamento Materno, Fonoaudiologia

## Abstract

**Purpose:**

To analyze the effects of lingual frenotomy on the breastfeeding of infants, based on the electrical activity of the masseter and suprahyoid muscles and assessment of the breastfeeding.

**Methods:**

Observational study developed between October 2017 and June 2018 with a sample of 20 newborns and infants who attended a dental clinic and were diagnosed with ankyloglossia. Another 20 were excluded for meeting some of the following exclusion criteria: babies more than 6 months old, who were not on exclusive or mixed breastfeeding, who had other clinical impairments that interfered with breastfeeding, who had other foods introduced into their diet, who had neurological changes and/or craniofacial deformities, and/or who did not finish all the stages of the study. Breastfeeding was assessed with the UNICEF Breastfeeding Assessment and Observation Protocol, while the muscle electrical activity was assessed with the Electrical Activity Assessment Protocol for the Masseter and Suprahyoid Muscles in Newborns During Breastfeeding. The same speech-language-hearing therapist conducted the two assessments both before the conventional frenotomy and 7 days after it.

**Results:**

The signs suggestive of breastfeeding difficulties changed 7 days after the surgery, with a p-value ≤ 0.002 for general observation of the mother, position of the infant, latch, and sucking. The maximum voluntary contraction of the masseter was the only integral parameter with a difference, as the electrical activity had decreased.

**Conclusion:**

Behaviors favorable to breastfeeding increased 7 days after the frenotomy in all the breastfeeding assessment categories, whereas the electrical activity of the masseter decreased.

## RESUMO

**Objetivo:**

Analisar os efeitos da frenotomia lingual na amamentação de recém-nascidos, com base na atividade elétrica dos músculos masseter e supra-hióideos e avaliação da mamada.

**Método:**

Estudo observacional, desenvolvido entre outubro de 2017 e junho de 2018. Amostra de 20 recém-nascidos e lactentes de uma clínica odontológica, diagnosticados com anquiloglossia; 20 foram excluídos por se encaixarem em algum dos critérios de exclusão: ter mais de seis meses de vida, não fazer aleitamento materno exclusivo ou misto, apresentar outro comprometimento clínico que interfira na amamentação, ter iniciado a ingestão de outros alimentos, alterações neurológicas e/ou deformidades craniofaciais e não ter finalizado todas as etapas do estudo. A avaliação da amamentação seguiu o Protocolo de Observação e Avaliação da Mamada da UNICEF, e a avaliação da atividade elétrica muscular o Protocolo de Avaliação da Atividade Elétrica dos Músculos Masseter e Supra-hióideos em Recém-Nascidos Durante a Alimentação, ambas realizadas pela mesma fonoaudióloga antes da frenotomia convencional e sete dias após.

**Resultados:**

Os sinais sugestivos de dificuldade na amamentação mudaram sete dias após a cirurgia, com valor de p≤0,002 para a observação geral da mãe, posição do bebê, pega e sucção. Houve diferença apenas no parâmetro integral da Contração Voluntária Máxima do masseter, com diminuição da atividade elétrica.

**Conclusão:**

Sete dias após a frenotomia observou-se aumento de comportamentos favoráveis ​​à amamentação em todas as categorias de avaliação da mamada e diminuição da atividade elétrica do masseter.

## INTRODUCTION

Ankyloglossia is a congenital oral anomaly that occurs when remaining embryological tissues, which should have undergone apoptosis in the embryonic development, remain on the lower surface of the tongue, restricting its movements^(1)^. Hence, ankyloglossia results from an embryologic failure in the process of separating the tongue from the floor of the mouth^(2)^. Its treatment is always surgical^(3)^, as the attachment of the frenum to the tongue and the floor of the mouth does not change over time^(4)^ and its histological constitution does not allow it to rip on its own or be stretched with exercises^(4)^.

In Brazil, to early diagnose and treat ankyloglossia, law no. 13.002/14 makes it mandatory for all hospitals and maternities nationwide to perform the lingual frenum assessment protocol - popularly known as the “Tongue-Tie Test” - before the baby is discharged^(5)^.

The impact of ankyloglossia on orofacial functions of sucking, swallowing, breathing, chewing, and speaking is an important public health issue. Its early diagnosis, associated with adequate procedures, are indispensable factors to minimize the impairment in the development of the stomatognathic system^(6)^.

Studies^(7-9)^ conducted through ultrasound have shown the importance of tongue movements to extract milk in breastfeeding. When making a dynamic analysis to explore the tongue movements in relation to the jaw during breastfeeding, a study observed constant oscillations in the jaw and tongue, without the movement of the buccinator muscles. It described that the movement of the anterior part of the tongue was predicted by oscillations in the jaw, while the posterior part of the tongue was curved to make swallowing easier and to coordinate with breathing^(10)^.

The authors made a rigid record of the jaw with the tongue contours during the breastfeeding movements and observed that the anterior part of the tongue was between the nipple-areola complex and the lower lips, continuously moving along with the jaw, in a slight posteroanterior direction as the jaw went downward, and vice-versa. Thus, this anterior motility is in agreement with the baby’s tongue pressure reflex, which is triggered when the lips are touched and the tongue stretches out of the mouth, enabling the baby to feed on the breast or bottle. This can be a matter of great concern in babies with ankyloglossia^(10)^.

Other studies have used surface electromyography to analyze the electrical activity in the muscles involved in the baby’s feeding. When analyzing preterm newborns without lingual frenum changes, they observed that for breastfeeding there is a balance in the electrical activity between the suprahyoid and masseter muscles. Also, there was no difference in muscle activity between the temporal, masseter, and buccinator muscles^(11)^.

A study conducted with 235 infants analyzed the electrical activity in the suprahyoid muscles based on the attachment of the lingual frenum to the tongue and the roof of the mouth while breastfeeding. They observed greater electrical activity in the muscle while sucking from the mother’s breast when the lingual frenum was attached to the middle third of the tongue and was visible from the sublingual caruncles. There was less electrical activity in the suprahyoid muscles while sucking from the mother’s breast, with the lingual frenum attached to the tip of the tongue and visible from the lower alveolar crest. They also observed the muscle’s greater electrical activity in babies with thin lingual frenum, and a pace of various suctions with short pauses, feeding efficiency adequately coordinated and balanced with the functions of sucking, swallowing, and breathing, with no signs of stress, no biting of the nipple, and no clicking the tongue^(12)^.

Given the above, developing studies in babies diagnosed with ankyloglossia and submitted to lingual frenotomy is of great relevance. The electrical activity makes it possible to understand its impacts both on the behavior of the muscles involved in breastfeeding and on the functioning of breastfeeding.

Therefore, this study aimed to analyze the effects of lingual frenotomy on full-term newborns’ breastfeeding, based on the electrical activity of the masseter and suprahyoid muscles, as well as the observation and assessment of breastfeeding.

## METHODS

This was an observational, analytical, cross-sectional study, conducted in accordance with the principles of the Declaration of Helsinki, approved by the Human Research Ethics Committee of the Health Sciences Center of the Federal University of Pernambuco (CEP50670-901/UFPE), under certificate no. CAEE 71186517.0.0000.5208, and evaluation report no. 2.283.175.

The study was developed between October 2017 and June 2018, with the mother-child dyad. They were receiving care at the teaching clinic of the Federal University of Pernambuco, where interdisciplinary services are offered, with dental and speech-language-hearing assistance.

This study approached a convenience sample, whose inclusion criterion was the baby’s having been diagnosed with ankyloglossia through the lingual frenulum evaluation protocol for infants^(6)^ (Tongue-Tie Test). The exclusion criteria were as follows: babies more than 6 months old, who were not on exclusive or mixed breastfeeding, who had other clinical impairments that interfered with breastfeeding, who had other foods introduced into their diet, who had neurological changes and/or craniofacial deformities, and/or who did not finish all the stages of the study.

The babies were first submitted to the lingual frenum assessment. They were considered apt to participate in the study when the items in the anatomo-functional assessment of the clinical examination summed seven or more - which demonstrated the interference of the frenum in the tongue movements. The same speech-language-hearing therapist, who had experience in applying these assessments, conducted the two of them both before the conventional frenotomy and 7 days after it.

The babies’ mothers were informed about all the procedures, risks, and benefits, and signed the informed consent form.

The mother-child dyads were assessed in two moments - the first before lingual frenotomy, by the teaching clinic’s dental team; the second, seven days after the surgery. A dentist performed the conventional frenotomy surgical procedure. Although hospitals are legally required to assess the lingual frenum, the study participants spontaneously sought the dental clinic for assessment. The potential study participants were instructed to return after 7 days for frenum assessment and, following the diagnosis, for breastfeeding assessment and electromyography. Once the breastfeeding assessment and electromyography were finished, the patient was submitted to conventional frenotomy; then, the mother was instructed to return after another 7 days for the last breastfeeding assessment and electromyography.

Two assessments were carried out: the breastfeeding assessment, according to the Breastfeeding Assessment and Observation Protocol^(13)^; and the assessment of the muscle electrical activity, through the Electrical Activity Assessment Protocol for the Masseter and Suprahyoid Muscles in Newborns During Breastfeeding^(14)^. Both were conducted simultaneously.

The Breastfeeding Assessment and Observation Protocol aims to identify signs favorable to breastfeeding and signs suggestive of difficulties (unfavorable). It is considered the gold standard in breastfeeding assessment, as it is ample and comprehensive, assessing not only the sucking but also aspects of the mother-baby interaction^(15)^. It is divided into four categories: general observation of the mother, position of the baby, latch, and sucking. Each category has four favorable and four unfavorable signs. Thus, breastfeeding is classified as good (0 to 1 unfavorable sign), average (2 unfavorable signs), or bad (3 to 4 unfavorable signs).

To classify breastfeeding as good, the mother must be comfortably seated, with her feet on the floor. This makes the baby’s positioning easier and enables its mouth to be on the same level as the areola. The baby’s body must be supported completely facing its mother and close to her, the head and spine aligned in a straight line, on the same axis. The baby’s mouth must be turned to the nipple so that it can enclose most of the areola. The chin must touch the mother’s breast and the mouth must be wide open; the lips must be turned outward; the upper part of the areola must be more visible than the lower, and the cheeks must be rounded^(15)^. The mothers were thus verbally instructed regarding the adequate breastfeeding position latch to improve suction. If the mother did not understand the instructions, the dyads received practical help. Both the verbal instructions and practical help (when necessary) took place immediately before the breastfeeding assessment.

The Electrical Activity Assessment Protocol for the Masseter and Suprahyoid Muscles in Newborns During Breastfeeding^(14)^ was conducted with the eight-channel Miotool Face USB, connected to a notebook via USB cable. The MiotecSuite 1.0 software was used, which is a system for acquiring data with the possibility of selecting eight independent gains per channel; in this case, the gain of 1000 was used; the band-pass filter of 20 to 500 Hz; externally USB-rechargeable NiMH battery, lasting approximately 60 hours - i.e., it works regardless of connection to an electrical outlet or a computer. Also, two sEMG sensors with claw connections were used; reference wire (ground) and calibrator. All the abovementioned material is manufactured by MIOTEC®.

Disposable surface neonatal electrodes, manufactured by MAXICOR®, were used. These were made of adhesive foam, silver rivets (Ag/AgCl), solid cellulose conductive gel, and PVC protective film.

**Figure 1:**
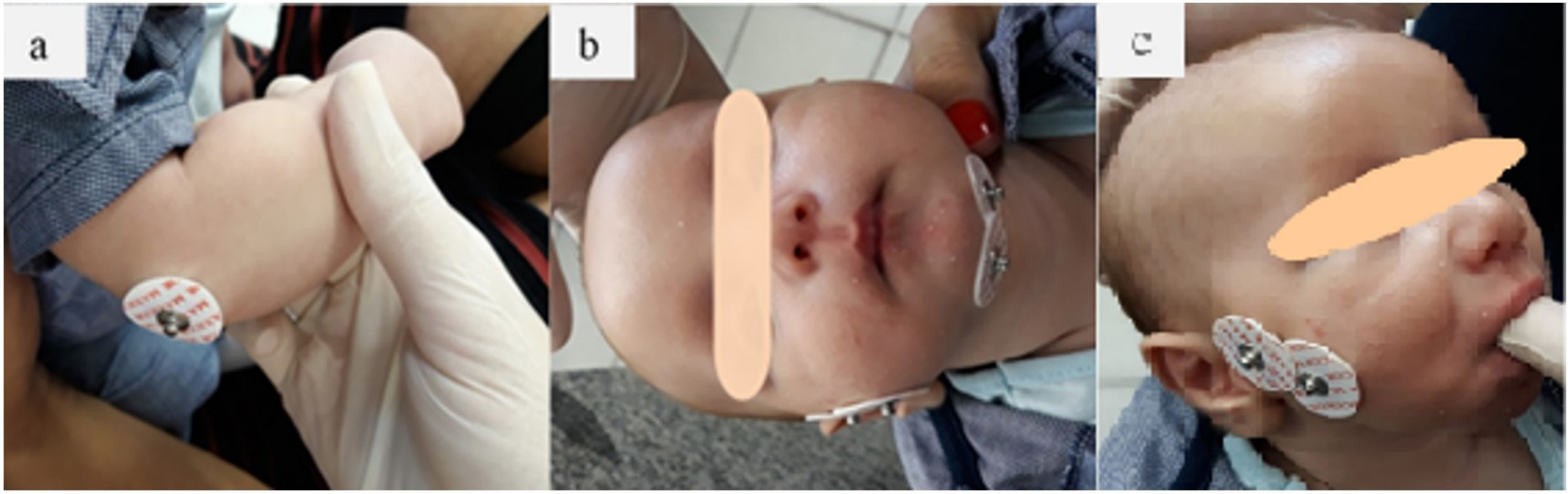
Image showing the placement of the electrodes: (a) in the olecranon of the ulna; (b) in the region of the masseter muscle, and the region of the suprahyoid musculature in babies submitted to lingual frenotomy; (c) electrodes in the region of the masseter muscle.

The preparation for the examination followed the cleaning procedures, as well as the placement of electrodes and sensors. The configuration was verified, and the two channels were enabled in the MiotecSuite 1.0, arranged as follows: Channel 1 - Region of the masseter muscle; Channel 2 - Region of the anterior suprahyoid muscle (Figure 1). The other unused channels were duly disabled. Once this stage was finished, the electromyography was started. The infant was awake during the procedure, and the assessment was made when they were hungry and breastfeeding. Also, the mother was instructed on the adequate latch and breastfeeding position. They were not undergoing speech-language-hearing follow-up or participating in the human milk bank. Some mothers had red, engorged, and/or painful breasts. After the surgery, the mothers were instructed to freely breastfeed on demand.

The electromyographic assessment had two stages. First, the electromyographic signals were picked up for normalization, through the signal of maximum resisted reflex activity (MRRA) of the masseter muscle. It used the bite reflex during the stimulation of the lateral alveolar region to maintain occlusion; and of the suprahyoid muscles, using the sucking reflex during non-nutritive sucking stimulation. Both stimuli were done with a gloved finger, for five seconds. For the analysis, the mean in μV (microvolts) of the three middle seconds of the test was used; that is the moment when the signal is most stabilized. The second stage was the muscle electrical activity (MEA), the mean of the action potentials of the motor units of a muscle group, during breastfeeding, for one minute, counted after the latch was stabilized.

For the presentation and interpretation of the signal, the MiotecSuite 1.0 was used. It was responsible for transforming the raw signal in RMS (Root Mean Square), which represents, in the digitalized signal, the result of the square root of the mean of the square of the instant amplitudes of the signal of the electromyographic tracing recorded, expressed in μV. The results found were transcribed to the Data Computation Sheet of the Electromyographic Assessment of the Newborn’s Feeding^(14)^.

The electromyographic signal was analyzed considering as reference value (100%) the mean in μV of three repetitions of each stimulus conducted in each muscle in the MRRA task. At this moment, an expressive number of muscle fibers is voluntarily recruited. All the other signals were analyzed in percentages of this reference value, for each baby. Such a normalization follows the recommendation from the International Society of Electrophysiology and Kinesiology^(16)^.

The percentage was calculated using the formula (X/MVC) x 100, in which X is the MEA mean during breastfeeding (μV), and MVC (maximum voluntary contraction) refers to the value corresponding to the MEA mean in MVC (μV).

The electromyographic signal was picked up for one minute when the mean number of peaks was calculated, as well as the mean time in between them. For such, each peak was delimited by the highest number of signals, corresponding to the upward phase plus the downward phase of the movement.

The statistical analysis was done in the Statistical Package for the Social Sciences (SPSS) software, version 20.0, using the Kolmogorov-Smirnov test to verify the normality of the data, besides the descriptive statistics with mean and standard deviation (SD). The Wilcoxon test was used to verify the differences between pre- and post-frenotomy, the Mann-Whitney test was used to verify the effects of the type of breastfeeding on MVC, and the Fisher exact test was used to analyze the association between the type of breastfeeding and the breastfeeding observation variables. For all tests, the significance level was set at 0.05%.

## RESULTS

Of the 40 babies diagnosed with ankyloglossia, 20 were excluded because their mothers did not return for the last assessment, and thus the infants did not finish all stages of the study. Hence, the sample comprised 20 mother-child dyads - 12 (60%) of the babies were males, and eight (40%) were female; their mean age at diagnosis was 51.6 days - a minimum of 13 and a maximum of 128 days (Table 1).

**Table 1:** Characteristics of the lingual frenum, Tongue-Tie Test score, and type of breastfeeding of the 20 infants assessed

**Sex**	**Age (days)**	**Type of breastfeeding**	**Frenum Insertion on the Sublingual (Ventral) Surface of the Tongue**	**Frenum Thickness**	**Lip Posture at Rest**	**Tongue-Tie Test Score**
**M**	13	Exclusive	Between the middle third and the tip of the tongue	Thin	Open	7
**M**	37	Mixed	Between the middle third and the tip of the tongue	Thin	Parted	7
**M**	21	Mixed	Between the middle third and the tip of the tongue	Thin	Open	12
**M**	91	Mixed	Between the middle third and the tip of the tongue	Thin	Open	8
**M**	128	Exclusive	Tip of the tongue	Thin	Open	9
**M**	30	Exclusive	Tip of the tongue	Thin	Open	12
**M**	52	Mixed	Tip of the tongue	Thin	Open	9
**M**	97	Exclusive	Between the middle third and the tip of the tongue	Thin	Parted	7
**M**	20	Mixed	Tip of the tongue	Thin	Open	10
**M**	67	Mixed	Tip of the tongue	Thin	Parted	9
**M**	55	Exclusive	Tip of the tongue	Thin	Parted	9
**M**	51	Exclusive	Between the middle third and the tip of the tongue	Thin	Parted	7
**F**	51	Mixed	Between the middle third and the tip of the tongue	Thin	Open	7
**F**	27	Mixed	Between the middle third and the tip of the tongue	Thin	Open	8
**F**	54	Mixed	Between the middle third and the tip of the tongue	Thin	Parted	7
**F**	63	Exclusive	Between the middle third and the tip of the tongue	Thin	Parted	10
**F**	22	Exclusive	Between the middle third and the tip of the tongue	Thin	Parted	7
**F**	62	Mixed	Between the middle third and the tip of the tongue	Thick	Open	9
**F**	61	Exclusive	Tip of the tongue	Thin	Open	9
**F**	30	Exclusive	Between the middle third and the tip of the tongue	Thin	Parted	7

Caption: M: male; F: female

According to the mothers’ reports, 10 (50%) out of the 20 infants were on exclusive breastfeeding, while the other 10 (50%) had infant formula after breastfeeding. They reported that the formula was given in baby bottles (six infants), baby spoon bottles (one infant), and sippy cups (three infants) right after natural breastfeeding.

Regarding the variables related to breastfeeding observation and assessment, the signs suggestive of difficulty in breastfeeding were different seven days after the surgery, with a p-value ≤0.002 for general observation of the mother, position of the baby, latch, and sucking. Fewer signs suggestive of difficulty in breastfeeding were observed then (Table 2).

**Table 2:** Comparison of the breastfeeding observation and assessment of 20 babies before frenotomy and seven days after it, during breastfeeding

**Variables**	**(Pre-frenotomy)**		**(Post-frenotomy)**		**p-value**
	**Median**		**Median**		
	50^a^ (25^b^ - 75^c^)		50 (25 - 75)		
General observation of the mother	2	(1 - 2)	1	(1 - 1)	<0.001*
Position of the baby	3	(1 - 3)	1	(1 - 1)	0.001*
Latch	3	(3 - 3)	1	(1 - 1)	<0.001*
Sucking	1	(1 - 2)	1	(1 - 1)	0.002*

Wilcoxon test

* p-value < 0.05; ^a,b,c^first, second, and third quartile, respectively.

Regarding the variables related to the electromyographic findings, the results revealed that there was a difference only in the integral parameter of the masseter MVC. The electrical activity had decreased seven days after the lingual frenotomy (Table 3).

**Table 3:** Comparison of the electromyographic findings of 20 babies before frenotomy and seven days after it, normalized in relation to the MVC^a^, during breastfeeding

**Variables**	**Pre-frenotomy**	**Post-frenotomy**	**p-value** *
	**Mean ± SD**	**Mean ± SD**	
Masseter - mean MVC^a^ (%)	25.52 ± 14.63	20.84 ± 12.22	0.235
Masseter - integral MVC^a^ (µV/s)	511.1 ± 293.7	361.1 ± 190.8	0.027*
Suprahyoid - mean MVC^a^ (%)	26.40 ± 9.10	27.81 ± 11.78	0.301
Suprahyoid - integral MVC^a^ (µV/s)	789.5 ± 1366.3	479.3 ± 261.0	0.161

Wilcoxon test

a MVC: maximum voluntary contraction

* p < 0.05

Caption: SD: standard deviation; µV/s: microvolts/seconds; % percentage

The Mann-Whitney test showed that the type of breastfeeding in this study sample had no effect on either masseter MVC (U = 40.5; p > 0.473) or suprahyoid MVC (U = 41.0; p > 0.49). Moreover, Fisher exact test showed no association between the type of breastfeeding and the mothers’ general observation (X^(2)^_(1)_ = 1.25; p = 0.71), babies’ position (X^(2)^_(1)_ = 1.97; p = 0.35), latch (X^(2)^_(1)_ = 1.05; p = 1.0), or suction (X^(2)^_(1)_ = 0.90; p = 0.80).

## DISCUSSION

The study was limited by the number of data lost due to incomplete assessments. This may have influenced the statistical analysis. However, the reliability of the data and the adequate analysis given the sample size validated the results. Furthermore, the study approached as yet unanswered hypotheses in the literature, which are of great importance to ascertain the effects of lingual frenotomy in breastfeeding.

Agreeing with other studies, ankyloglossia in the sample studied here occurred more often in males^(17,18)^. Nevertheless, some studies observed sample characteristics similar between the genders^(7,18)^, which does not define the diagnosis of ankyloglossia as a predominantly male characteristic.

The mean age of the babies assessed was 51.6 days, which disagrees with the mean age seen in other studies^(18)^. This can be justified by the individual characteristics of each service. A bibliographic survey conducted in 2016, which included articles published from 2000 to 2014, demonstrated that the population studied was in the age range from 0 to 30 days^(18)^.

The early age for diagnosis and correction of ankyloglossia, mentioned in the literature, is seen as a factor that can help reduce early weaning during breastfeeding. Also, it can minimize changes in the development and performance of the babies’ orofacial fucntions^(9)^. Thus, for the baby to be diagnosed in the first days of life and the procedures to start early, aiming for the improvement of both the mother’s and the baby’s quality of life, it is necessary to have an interdisciplinary assessment, involving a speech-language-hearing therapist, dental surgeon, and/or pediatrician^(9)^. The minimum age in this study sample was 13 days, and the maximum age was 128 days, with a mean age of 51.6 days.

The breastfeeding observation and assessment protocol revealed, in the first assessment, that the median of the general observation of the mother was average. It indicated two unfavorable behaviors in the breastfeeding observation; the position of the baby was bad, varying the quartiles between one and three behaviors unfavorable to breastfeeding; also, the latch was bad, though with a prevalence of two and three unfavorable behaviors; sucking, in its turn, was observed as good, although the quartiles were of one to two unfavorable behaviors.

In a study with a sample of 165 babies, 61 (36.96%) were diagnosed with lingual frenum changes. This revealed that, even though the correlation between the frenum and sucking was low, babies with an abnormal lingual frenum are more likely to have inadequate sucking^(18)^.

Despite the methodological differences, it is possible to identify lingual frenum changes as important aspects when assessing babies with difficulties in breastfeeding. This highlights the interference in the breastfeeding process and the mother-child relationship during breastfeeding.

The literature^(19,20)^ indicates that other factors are also associated with breastfeeding difficulties. The study by Santos et al.^(19)^ showed that adolescent mothers had greater difficulties dealing with the newborns’ behavior in breastfeeding than adult or late mothers. Alves et al.^(20)^ demonstrated the existence of a relationship between the posture of the mother-infant dyad, poor quality of breastfeeding, and difficulties with mandible movements. The study points out the need for instructing and preparing mothers for breastfeeding, as well as adjusting the sucking patterns in infants.

Another aspect to consider is the breast and nipple status. In the dyads studied, 10 mothers had red, engorged, and/or painful breasts. Alves et al.^(20)^ point out that such a condition may interfere with the sucking pattern, latch, and tongue movement in infants, leading to early weaning.

It is important to mention that different circumstances contribute to the difficulty of breastfeeding. Among them, there are neurological changes and/or craniofacial deformities. Nonetheless, these characteristics were encompassed in this research’s exclusion criteria, as ankyloglossia could be secondary to some type of genetic syndrome^(21)^.

Even though there is still no consensus in the literature regarding the effectiveness of lingual frenotomy in the treatment of ankyloglossia, the results observed in this study demonstrate an increase in favorable behaviors seven days after the lingual frenotomy. Consequently, there was a decrease in unfavorable behaviors. The median in all categories (general observation of the mother, position of the baby, latch, and sucking) was a favorable behavior after the surgery. Similar results were also found in other studies^(22-27)^, which made it possible to obtain a prognosis favorable to maintaining breastfeeding in babies without lingual frenum changes.

For quantitative muscle findings, the study investigated the muscle electrical activity through surface electromyography of the masseter muscle - as it actively participates in the functions of sucking, protruding, elevating, and retracting the jaw - and of the suprahyoid musculature - which also participates directly in sucking, moving and stabilizing the jaw, and moving the tongue. In general, the suprahyoid muscles move not only the jaw and the hyoid bone but also the floor of the mouth. Both regions are easily found, making it easier to place the electrodes^(12)^.

The change observed in the results seven days after lingual frenotomy - when analyzing the records of the mean MVC in percentage and the integral of the masseter muscle in relation to the MVC - shows a modification in breastfeeding patterns through the decreased electrical impulses^(11)^. Consequently, there was less energy in the signal, corroborating a study conducted in 2014. It showed that the masseter participates actively in the jaw movement when breastfeeding, especially in elevating and protruding the jaw^(11)^.

In this study, it is possible that when the baby still had their tongue movement restricted by ankyloglossia, the participation of the masseter was overloaded at the first moment to adapt the jaw movements.

Considering the attachment of the frenum to the middle third/sublingual caruncles (considered normal) and to the tip of the tongue/lower alveolar crest (considered abnormal), the reports in the literature describe a greater activity in the suprahyoid musculature during breastfeeding of babies with normal frenum^(12)^. In this study, there was no difference in the percentage of the electrical activity of this musculature, comparing the pre- and post-frenotomy moments. Neither was there any difference between the number of suctions (peaks) and the pause in between the sequences of sucking (mean of the peaks’ time) in the record of the masseter muscle electrical activity - contrary to what the literature describes^(3)^. However, this study assessed breastfeeding seven days after the procedure, and the modifications in sucking patterns may not have concluded by then.

With the pattern modification after frenotomy, the tongue may perform the movement necessary to adequately extract the milk from the mother’s breast and swallow it. Thus, the masseter muscle would not be overloaded^(28)^, and the number of suctions and the pause in between suctions would be maintained without causing so much muscle effort.

This information shows the importance of assessing newborns early to identify changes and seek to solve them as soon as possible. The tongue has direct and important action in different stomatognathic functions and influences eating, chewing, swallowing, and speaking^(29)^. Therefore, this change can cause a functional and social loss in all phases of life.

This study furnishes more indications of the impact of ankyloglossia, as well as the influence of surgical treatment on these babies’ quality of life. Such changes are already observed after the procedure.

It is necessary to conduct studies with larger samples, using other objective examinations, such as ultrasound, associated with the qualitative assessment of breastfeeding to clarify the importance of the correct indication of frenotomy, besides helping with scientific evidence.

## CONCLUSION

The data obtained in this study show that babies with ankyloglossia have behaviors unfavorable to breastfeeding, related to the mothers’ general observation, babies’ position, latch, and suction. Also, there was an increase in behaviors favorable to breastfeeding in all categories seven days after the conventional frenotomy. The type of breastfeeding revealed no effect on MVC variables nor any association with breastfeeding observation variables. The decrease in the electrical activity of the masseter muscle was also observed; however, the decrease in the electrical activity of the suprahyoid muscles had no significant statistical difference after the surgery.
